# Mitochondrial genetic effects mediate the response to stress through development, but not adult metabolic rate in *Drosophila melanogaster*

**DOI:** 10.1242/jeb.253155

**Published:** 2026-10-20

**Authors:** Avishikta Chakraborty, Enrique Rodríguez, Stefano Bettinazzi, Stefania Stroila, Nick Lane, M. Florencia Camus

**Affiliations:** Research Department of Genetics, Evolution and Environment, University College London, Gower Street, London WC1E 6BT, UK

**Keywords:** Mitonuclear interactions, *Drosophila melanogaster*, Mitochondria, Diet, Metabolic stressors, Early-life development, Metabolic rate

## Abstract

Energy expenditure is fundamental to physiology, behaviour, ecology and life history, yet the mechanisms that regulate metabolic rate remain poorly understood. At the cellular level, incompatibilities between the maternally inherited mitochondrial genome and the nuclear genome can impair energy production, signalling and gene expression, with potential to disrupt a wide range of physiological processes. However, how these often-subtle genomic mismatches influence whole-organism traits such as metabolic rate, activity and fitness remains unclear. Here, we generated mitonuclear-mismatched fly lines to test how early-life dietary and metabolic stress affect larval and adult physiology. Our results revealed sex- and line-specific physiological effects, with larval development, survival and female fertility strongly contingent on the haplotypes and treatment, while adult resting metabolic rate and activity were not influenced by mitochondrial haplotype, or by developmental stress.

## Teaser

**Summary:** Subtle mitochondrial genetic differences reshape developmental responses, survival and fertility under metabolic stress. Adult metabolic rate, however, remains remarkably robust to metabolic stress.

## INTRODUCTION

Mitochondria produce most of the energy of the cell in the form of ATP through oxidative phosphorylation (OXPHOS) and do so by relying on the correct function of enzymatic complexes whose subunits are encoded by two distinct genomes. Thirteen polypeptides are encoded by the mitochondrial DNA (mtDNA), while the rest are encoded in the nucleus (nDNA). Altering the co-adapted combinations of mtDNA and nDNA products (mitonuclear interactions) has been shown to influence multiple processes, from OXPHOS assembly and function to signalling via reactive oxygen species (ROS), organismal growth, reproduction, development, lifespan and nutritional preference (Bettinazzi et al., 2025, 2024; Camus and Inwongwan, 2023). This evidence often comes from studies looking at strong mitonuclear incompatibilities between different populations or laboratory-evolved lineages; however, more subtle mismatches between the two genomes [in one or a few single-nucleotide polymorphisms (SNPs)] have received comparatively less attention (Camus et al., 2023).

The effects of mitochondrial and nuclear genomes on phenotypic traits expressed at different life stages (e.g. juvenile and adult stages) are well documented, particularly in the fruit fly *Drosophila melanogaster* (Aw et al., 2018; Rand and Mossman, 2020). However, little is known about whether these epistatic genetic effects are consistent or change across different life stages. Developmental nutrition and metabolic challenges (such as restricted diets or perturbations of glycolysis and β-oxidation) can expose cryptic genetic variation by altering energetic demands, thereby revealing how mitonuclear background shapes physiological outcomes (Dobson et al., 2023; Piccinini et al., 2021; Quirós et al., 2016; Rodríguez et al., 2021). Metabolic rate (MR) represents a central physiological trait through which these effects can be quantified. Given the central role of mitochondria in energy metabolism, an important question is whether variation in mitochondrial genotype can influence metabolism at the level of whole-animal MR (Metcalfe et al., 2023). One possibility is that adult MR, as an integrated physiological phenotype, remains relatively stable under moderate mitochondrial stress as a result of compensatory responses such as altered mitochondrial biogenesis, shifts in substrate use or behavioural adjustments in activity (Thoral et al., 2024). In contrast, development involves periods of intense biosynthetic demand, tissue differentiation and metabolic reorganisation, which may constrain compensatory capacity and increase vulnerability to mitonuclear mismatch. Consequently, phenotypic effects arising during development may weaken or change in expression by the adult stage (Moran et al., 2024; Mossman et al., 2017; Wolff et al., 2014).

Here, we used a *Drosophila* panel carrying distinct mitochondrial haplotypes on an otherwise isogenic nuclear background to test how developmental metabolic stress interacts with mtDNA genotype to shape physiological outcomes across life stages (Camus et al., 2023; Rodríguez et al., 2021). Our lines included wild-type (WT, *w*^1118^) with co-evolved genomes, COX carrying a single SNP from WT in the mitochondrial *COXII* gene, and BAR containing nine SNPs from WT in protein-coding genes of complexes I and IV. We exposed flies to metabolic stressors during development, including a high-protein diet (HP), the glutathione precursor *N*-acetyl-l-cysteine (NAC) to perturb redox balance (Samuni et al., 2013), and nicotinamide riboside (NR) to manipulate NAD^+^ metabolism (Katsyuba and Auwerx, 2017). We then quantified sex- and genotype-specific responses across life stages, measuring developmental timing, fertility, activity and adult resting metabolic rate (RMR). This design allowed us to determine (i) whether mitonuclear background conditionally modifies physiology under developmental stress, (ii) whether effects are sex specific, and (iii) whether adult RMR is sensitive or resilient to earlier mismatches and stress exposure. We found that development, fertility and activity depend strongly on mitochondrial haplotype and treatment, with clear sex- and line-specific patterns, whereas adult whole-organism RMR is not detectably influenced by mitonuclear background or developmental stress in our system. These results suggest that mtDNA genomes can generate pronounced, context-dependent phenotypes without necessarily manifesting as changes in adult RMR, highlighting stage-specific sensitivity and the potential for compensatory buffering across the life course.

## MATERIALS AND METHODS

### *Drosophila* lines and experimental treatments

A mitonuclear panel composed of three strains of *Drosophila melanogaster* was generated through a balancer chromosome crossing scheme (Clancy, 2008). All three fly lines shared a common *w*^1118-5,095^ nuclear genome, while differing only in their mitochondrial genomes (Rodríguez et al., 2021). The coevolved wild type (WT) strain carried the mtDNA variant coadapted with the *w*^1118-5,095^ nuclear background, whereas both COX and BAR strains were introgressed with a foreign mtDNA. Specifically, when compared with WT *w*^1118-5,095^ mtDNA, the COX strain harbours a fixed COIIG177S mtDNA mutation – a single non-synonymous SNP in cytochrome *c* oxidase subunit II (Patel et al., 2016), whereas the BAR strain mtDNA differs from WT by nine SNPs, primarily in protein-coding regions (Wolff et al., 2014). Lines were kept at approximate densities of 80–100 eggs per vial, with 10 replicate vials per strain. All three strains were maintained on a standard mix of 8 ml molasses–cornmeal medium (Table S4) per vial at a constant temperature of 25°C on a 12 h:12 h light:dark cycle with 50% humidity. Lines were provided with *ad libitum* live yeast sprinkles every generation to promote egg laying.

We exposed the fly larvae to four experimental treatments (see Table S4 for detailed composition). The control diet was the standard medium of molasses–cornmeal that the strains were maintained on, with a protein-to-carbohydrate ratio of 1:4. The three metabolic stressful treatments consisted of dietary modifications: (i) a high protein diet, in which the amount of yeast (used as a proxy for protein content) was increased to reach a yeast-to-carbohydrate ratio of 1:2; (ii) supplementation with *N*-acetyl-l-cysteine (NAC, Sigma A7250), a glutathione precursor which acts as an antioxidant (Samuni et al., 2013), used at a concentration of 5 g l^−1^ in the control food; and (iii) supplementation with nicotinamide riboside (NR, WZ Biotech, CAS: 1341-23-7), a precursor of NAD^+^ (Katsyuba and Auwerx, 2017), used at a concentration of 10 mmol l^−1^ in the control media. About 6 ml of all the treatments was dispensed into individual fly vials using a 10 ml syringe. For all the following assays, the three strains were developed from eggs to adults on one of the four dietary treatments, generating 12 line×treatment combinations. For the female reproductive fitness experiment, males were obtained from a lab-adapted population (LHm) and were raised in the same control conditions as our mitonuclear strains.

### Larvae developmental time and survival

Three small cages containing yeast-sprinkled standard molasses food in Petri dishes were set up per strain, using adults from ∼5–6 replicate vials of each strain. The flies were left to lay eggs overnight at 25°C. Egg-filled Petri dishes were later divided into small chunks and transferred into vials containing 6 ml standard molasses food. For each strain, we had 40 vials separated across two blocks, ∼6 generations apart.

The emerging adults were then housed in small oviposition chambers, which contained agar–grape juice media (Table S1). They were allowed to oviposit overnight, following which 30 eggs were picked into 6 ml food vials containing each of the four treatments. Twenty replicate vials were set up with 30 eggs each for the 12 line×treatment combinations. Development time was recorded as the time it took flies from each vial to eclose. The vials were checked and eclosion scored 3 times per day (09:00 h, 12:00 h and 17:00 h) until the last fly from each vial emerged.

### Adult female reproductive fitness

Reproductive fitness of the females was measured as the total number of offspring produced by groups of three virgin females from each strain developed in the four treatments, when mated with groups of three males from the control fly population (LHm strain). Egg picking was staggered by a day to mitigate the developmental delays caused by treatments, and virgin females were collected from each of the 12 line×treatment combinations and housed in control 6 ml molasses food vials for 3 days to reach sexual maturity. Males from the standard lab-adapted LHm population were developed in density-controlled standard molasses food vials and collected the same way. After reaching sexual maturity, groups of three virgin females were mated with three LHm males in control molasses food vials, sprinkled with yeast. For each of the line×treatment combinations, 35–40 replicate vials were set up. Mating groups were discarded after 72 h of laying. Total offspring produced were counted from each vial once all the eggs laid had hatched.

### Mass-specific metabolic rate and activity

We measured whole-organism RMR in adult flies using a MAVEn-FT flow-through system (Sable Systems International). After developing in each of the four treatments described above, eclosed flies from each strain and treatment condition were transferred to control food until the assay. Each biological replicate (‘sample’) consisted of a group of five flies (4–10 days post-eclosion) from a given line×sex×treatment combination. Flies were briefly anaesthetised with 5 ppm CO_2_ and weighed collectively (wet mass) using a Sartorius MCA6.6S-2S00-M Cubis^®^ II microbalance. Each group of five flies was then placed in a 6.5×0.5 cm chamber connected to a LICOR 850 CO_2_ analyser (LI-COR). CO_2_ production was recorded for 120 s at a flow rate of 30 ml min^−1^, and activity was simultaneously measured via infrared beam breaks. During each measurement cycle, 16 chambers were assayed in parallel. Each chamber (i.e. each biological replicate) was measured 5 times within a cycle (technical replicates), following an initial acclimation period of 30 min. For each line×treatment×sex combination, we measured approximately 12 biological replicates (i.e. 12 independent groups of 5 flies; ∼60 flies total), distributed across two experimental blocks separated by one generation. Measurements were conducted in two daily runs (10:00–16:00 h and overnight from 17:00 h).

### Statistical analyses

Data collected from all the above assays were analysed using R software version 4.3.0 (https://www.r-project.org/). For development time, survival and body mass, each mitochondrial strain, treatment, sex and their interaction were modelled as fixed effects. Block effects were accounted for in the model as a covariate, with replicate vial for random effects. A linear mixed model was used for the response variable for egg-to-adult development time and body mass, whereas for survival, a binomial general linear model was fitted with offspring and files that failed to develop (deaths) as the response variable.

For adult female fitness, the total number of offspring produced was used as a response variable, with mitochondrial strain, treatment and their interaction as fixed effects. We analysed offspring counts using a negative-binomial generalised linear model because the data were overdispersed relative to a Poisson distribution (variance=2491.8, mean=102.2).

MAVEn data files were processed using Sable Systems International software (ExpeData v.1.7), which extracted the 30 s window (within the 2 min reading) with the lowest CO_2_ production and corresponding activity values of individual flies. Linear mixed models were fitted to log_10_-transformed mass and log_10_-transformed absolute metabolic rate data using the *lmer* function of the *lme4* package (Bates et al., 2015). Mitochondrial line, treatment, mass, activity level, block, run and chamber were accounted for in all linear regression models. Data were also analysed with mean-centred log_10_-transformed mass and mean-centred activity rate as continuous covariates to determine treatment effects on RMR. Similarly, for adult activity as a response variable, a linear mixed model was fitted with mitochondrial strain, treatment, mass, sex and block. Supporting data are provided in Tables S1–S4.

## RESULTS AND DISCUSSION

### Mitonuclear genotype, sex and the environment interact to shape early-life phenotype

Developmental time and survival to adulthood were both shaped by mitonuclear background and dietary treatment (Fig. 1; Table S1). Significant interactions were detected between line×treatment (χ^2^=1040.45, *P*≤0.001), sex×treatment (χ^2^=42.61, *P*≤0.001) and line×sex (χ^2^=9.68 *P*=0.008) on developmental time (Fig. 1A), with significant main effects of line and treatment on survival, and an interaction trend between the two (χ^2^=6.58, *P*=0.037; χ^2^=9.88, *P*=0.02; χ^2^=12.20, *P*=0.058, respectively; Fig. 1B).

**Fig. 1.**
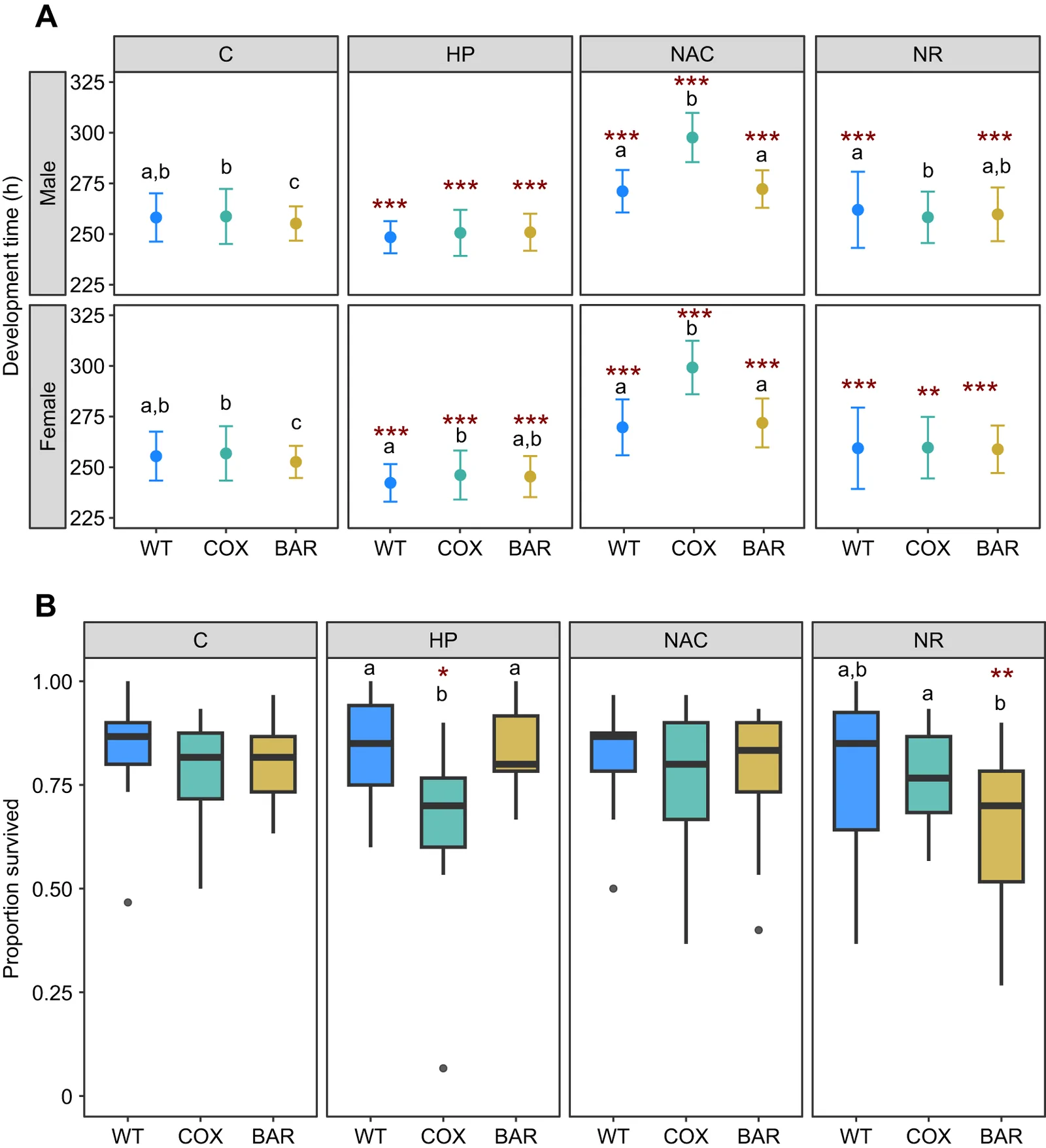
**Effect of dietary environment on develpmental time and survival, across mitonuclear lines and sex.** Development time (A) and proportion surviving (B) from egg to adult in each sex, treatment [control (C), high protein (HP), *N*-acetylcysteine (NAC), nicotinamide riboside (NR)] and mitonuclear line [wild-type (WT), COX and BAR]. Twenty replicate vials were set up with 30 eggs each for the 12 line×treatment combinations. Results are shown as means±s.d. in A and median, upper and lower quartiles and 1.5x the interquartile range in B. A linear mixed model was used for egg-to-adult development time; for survival, a binomial general linear model was fitted. Letters denote significant differences (*P*≤0.05) between lines within a treatment for each sex, while asterisks denote significant differences within a line, relative to the control diet (*P≤0.05, **P≤0.01, ***P≤0.001). There was a significant line×treatment (*P*≤0.001), sex×treatment (*P*≤0.001) and line×sex (*P*≤0.01) effect on developmental time, and a significant line and treatment effect on survival (both *P*≤0.05).

Under control conditions, female BAR flies developed significantly faster than both WT and COX flies (*t*_2927_=2.62, *P*=0.024 and *t*_2927_=3.77, *P*<0.001, respectively), consistent with prior observations in these lines (Rodríguez et al., 2021). HP diet accelerated development relative to control uniformly across all lines and sexes (*P*<0.001), as expected from TORC1-mediated anabolic growth (Wong et al., 2012). NAC prolonged development in all lines, but the effect was haplotype specific: COX flies were disproportionately delayed relative to both WT and BAR within the NAC treatment (by 29.44 and 26.44 h, respectively, *P*<0.001). This effect could be driven by the COXII substitution via perturbation of redox balance at complex IV, rendering this line especially vulnerable to glutathione-mediated ROS buffering during the energetically demanding larval period (Rodríguez et al., 2021; Samuni et al., 2013). NR also delayed development across lines and sexes relative to control (all *P*<0.001 except COX females where *P*=0.003), with the notable exception of COX males, which showed no significant response (*P*=0.99), revealing a sex-specific interaction with NAD^+^ manipulation unique to this haplotype.

Survival followed a complementary pattern: COX survival was significantly reduced under HP relative to controls (13% increase in mortality, *P*=0.04), whilst BAR suffered greater mortality under NR (14% increase, *P=*0.006), with the two mismatched lines diverging significantly within that treatment (14% difference, *P*=0.01). These haplotype-specific vulnerabilities to distinct metabolic stressors reinforce the view that even SNP-level mitonuclear mismatches can generate pronounced, context-dependent effects on early-life fitness (Gyllenhammer et al., 2020; Rodríguez et al., 2021).

### Phenotypic effects can persist through adulthood

After developing in one of the four environments, adults were transferred to control food, and both their reproductive output and body mass were measured (Fig. 2; Table S2). Again, we found a significant interaction between line and treatment on the total number of offspring produced by females (χ^2^_6_=18.69, *P*≤0.001; Fig. 2A; Table S2), and a main effect of treatment (χ^2^_3_=21.62, *P*<0.001; Table S2). In line with mounting evidence that mitonuclear discordance can severely impact reproductive fitness (Bettinazzi et al., 2024; Dobson et al., 2023; Meiklejohn et al., 2013; Rank et al., 2020), mismatched COX and BAR flies produced fewer offspring than co-evolved WT flies in control conditions (Tukey-adjusted *P=*0.0017 and *P=*0.0075, respectively; Table S2). Notably, early-life metabolic stress generally boosted the reproductive success of mismatched COX and BAR lines. Developing on an HP diet increased the fertility of COX (Tukey-adjusted *P*=0.006; Table S2) but not BAR flies (Tukey-adjusted *P=*0.25; Table S2). Developing on NAC and NR strongly increased offspring number in COX and BAR adults, relative to the control diet (Tukey-adjusted BAR: NAC, *P*=0.016; NR, *P*=0.005; COX: NAC, *P*=0.003; NR, *P*<0.001; Table S2). These effects were particularly pronounced under NR treatment, which increased offspring production by approximately 61% in BAR and 77% in COX flies, relative to controls. In contrast, the fertility of the co-evolved WT flies did not significantly respond to any dietary treatment relative to the control diet. However, offspring production was lower under HP than under NR conditions (Tukey-adjusted *P*=0.019; Table S2).

**Fig. 2.**
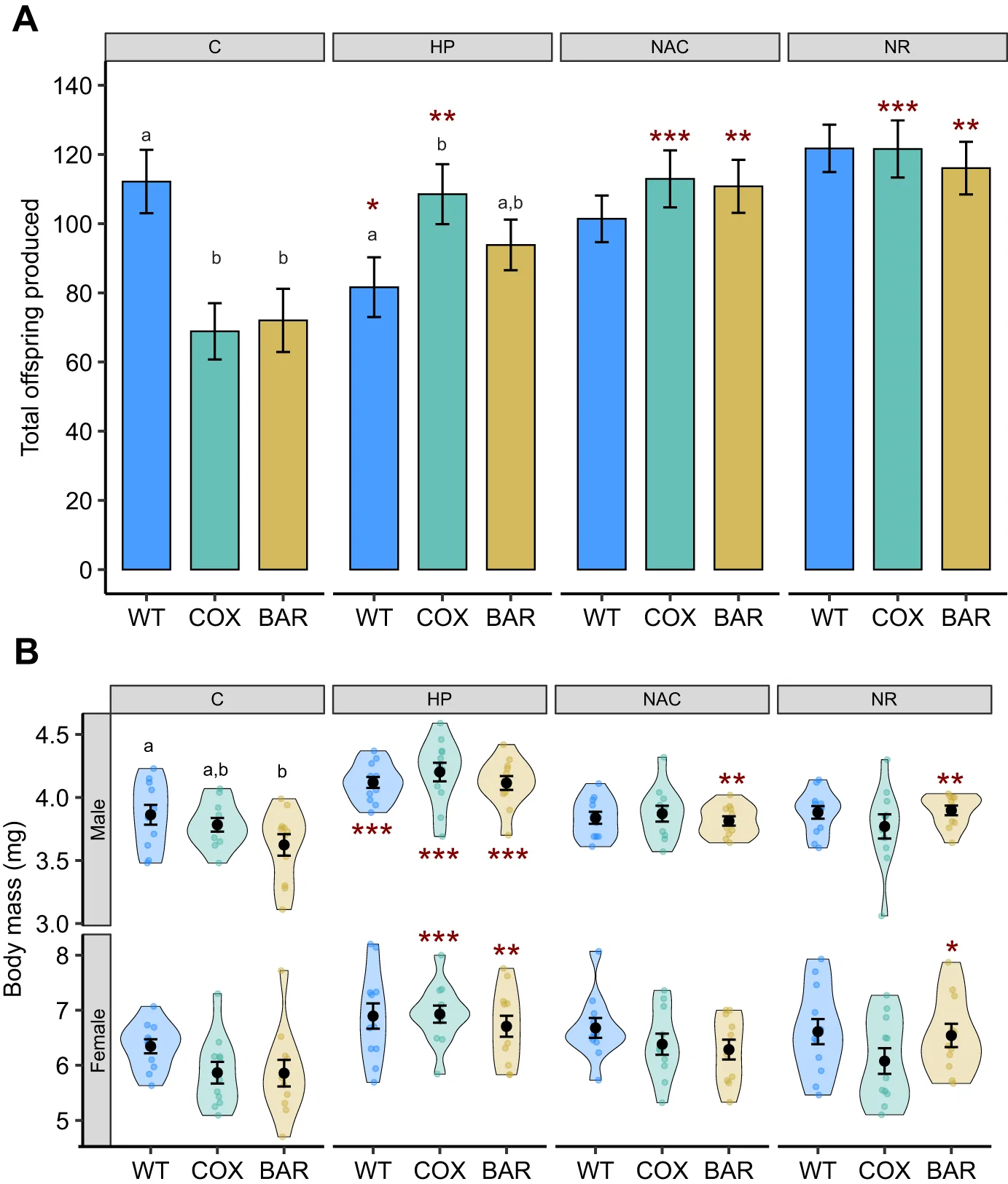
**Effect of developmental treatment on adult traits: female fitness and body size across mitonuclear lines.** The number of offspring produced by females (A) and adult body wet mass (B) of groups of five flies, shown for each sex, for the three mitochondrial haplotypes when developed in one of the four treatments. For adult reproductive fitness, 35–40 replicate vials were set up for each of the line×treatment combinations. For body mass, ∼12 biological replicates (i.e. 12 independent groups of 5 flies; ∼60 flies total) were set up for each line×treatment×sex combination, and this was distributed across two experimental blocks separated by one generation. For female fertility, a negative binomial generalised linear model was fitted. A linear mixed model was used for body mass. Results are shown as means±s.e.m. Letters denote significant differences (*P*≤0.05) between lines within a treatment for each sex, while asterisks denote significant differences within a line, relative to the control diet (*P≤0.05, **P≤0.01, ***P≤0.001). There was a significant line×treatment and treatment effect on female fertility (both *P*≤0.001) and a significant sex×treatment (*P*≤0.05), line (*P*≤0.05), sex (*P*≤0.001) and treatment effect (*P*≤0.001) on body mass.

Body mass showed significant main effects of sex (χ^2^=2137.96, *P*<0.001; Fig. 2B), treatment (χ^2^=63.76, *P*<0.001) and line (χ^2^=8.65, *P*=0.013), with a significant sex×treatment interaction (χ^2^=8.65, *P*=0.034). HP generally increased body mass across mitonuclear lines, although the magnitude of the response varied among sexes and genotypes (Fig. 2; Table S2). In females, HP increased body mass relative to the control treatment in BAR (Tukey-adjusted *P*=0.003) and COX (Tukey-adjusted *P*=0.0002) lines, whereas no significant effect was detected in WT females. NAC and NR effects were more line specific than those of HP, with significant mass increases observed primarily in BAR individuals, particularly under NR treatment in both males (Tukey-adjusted *P*=0.008) and females (Tukey-adjusted *P*=0.041). In contrast, NAC effects were generally weak and did not remain significant after Tukey correction. COX and WT individuals showed little evidence of significant body mass responses to NAC or NR treatments. The persistence of line- and sex-specific differences in fertility and body mass after transfer to common-garden conditions points to a lasting developmental imprint of early-life metabolic environment, consistent with growing evidence that mitonuclear background shapes physiological outcomes across life stages in a condition-dependent manner (Bettinazzi et al., 2025, 2024; Camus et al., 2023). Particularly striking is the paradoxical improvement in reproductive success of mismatched COX and BAR lines under NAC and NR despite these treatments imposing developmental costs, which we interpret as condition-dependent exposure of cryptic genotype×environment interactions whereby stress redirects resources toward adult reproductive investment (Dobson et al., 2023; Rodríguez et al., 2021). That such effects persist beyond metamorphosis suggests that redox- or NAD^+^-sensitive signalling during larval development can reprogramme germline allocation in ways that outlast the larval stage (Makwisa et al., 2026; Stefanatos et al., 2025), with the male-specific mass increases in BAR further reflecting known sex differences in the persistence of larval nutritional effects on adult phenotype across holometabolous insects.

### Metabolic rate in adulthood is resilient to mitonuclear×treatment interactions in development

In contrast with the life-history effects described above, whole-organism physiology showed little sensitivity to mitonuclear background. Developmental treatment significantly affected RMR in males (χ^2^=7.86, *P*=0.048), but not females, whereas activity remained unaffected by developmental treatment, mitonuclear background and their interaction in both sexes (Fig. 3; Table S3). This is notable given that our previous work on these very lines revealed lower mitochondrial respiratory output in mismatched COX and BAR adults under redox stress (Camus et al., 2023), suggesting that mitonuclear mismatch can affect mitochondrial function without propagating to the whole-organism metabolic level. The relationship between mitochondrial function and metabolic rate is not straightforward (Metcalfe et al., 2023; Salin et al., 2016; Thoral et al., 2024, 2026): bioenergetic dysfunction may be buffered through compensatory mechanisms such as altered substrate use or mitochondrial biogenesis, leaving RMR relatively stable despite underlying variation in cellular energetics.

**Fig. 3.**
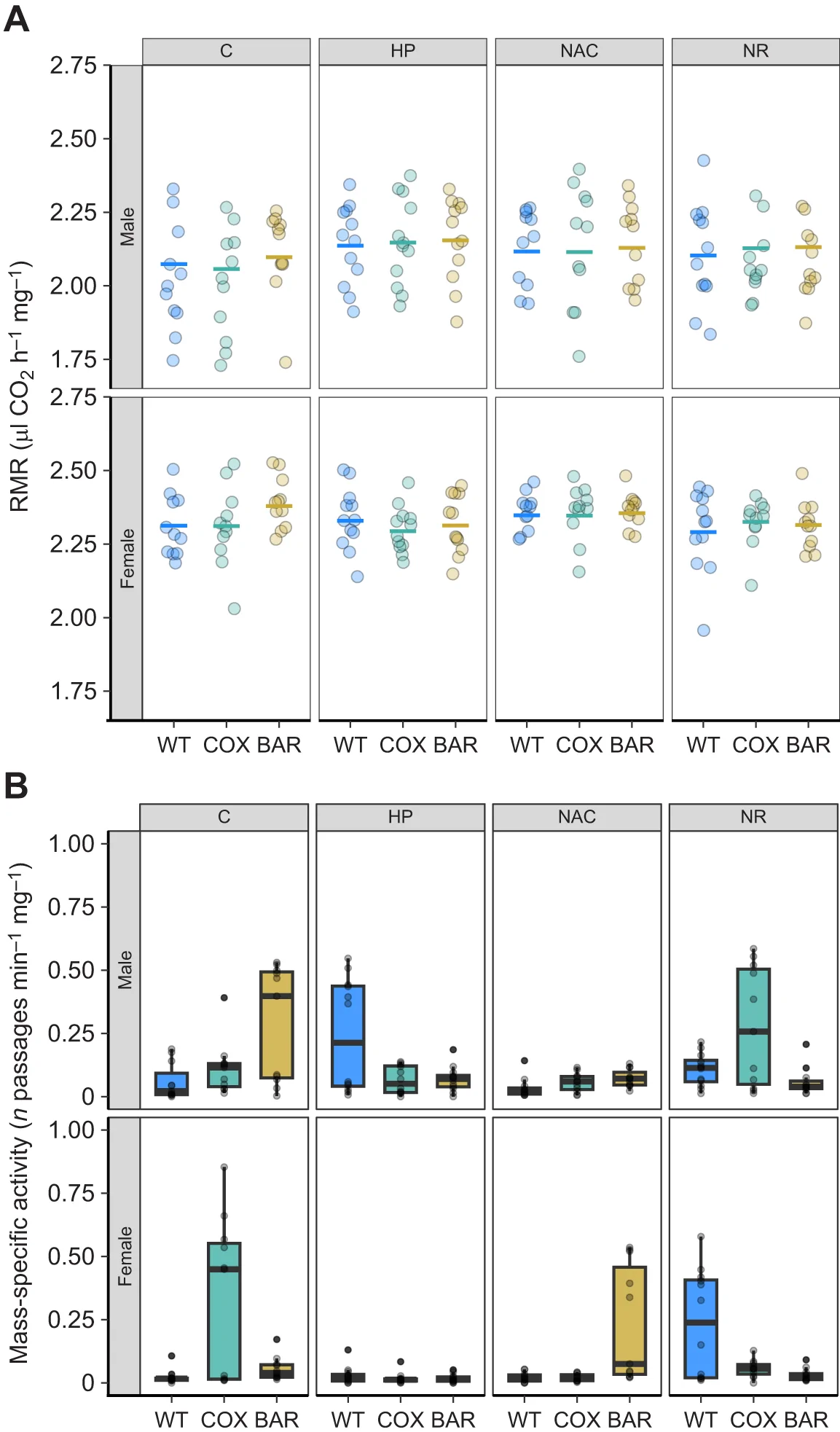
**Effect of developmental treatment on adult traits: resting metabolic rate (RMR) and mass-normalised activity, across mitonuclear lines and sex.** (A) Rate of CO_2_ production, as the measured *V̇*_CO_2__ values standardised to the mean log_10_-transformed mass and zero activity levels. Dashed lines are model-estimated predictions of group means, adjusted for covariates and random variables based on model parameter estimates for log_10_-transformed mass and activity rate (see Materials and Methods). (B) Activity recorded in the MAVEn respirometer and normalised to fly mass. For both RMR and activity, we measured ∼12 biological replicates (i.e. 12 independent groups of 5 flies; ∼60 flies total) for each line×treatment×sex combination, distributed across two experimental blocks separated by one generation. Linear mixed models were fitted for both. There was a significant treatment effect (*P*≤0.05) on RMR for males.

Understanding why some traits are more sensitive to mitonuclear interactions and early-life conditions than others remains challenging. Although interfering with protein-to-carbohydrate balance appears to have broadly similar effects on development regardless of mitonuclear background, the effects of perturbing redox balance are more context specific. NAC, as a cysteine donor supporting glutathione synthesis, can buffer ROS-sensitive developmental signals including the JNK, FoxO and insulin/IGF pathways (Quirós et al., 2016), potentially reshaping growth rates and tissue allocation in a genotype-dependent manner. NR, by elevating NAD^+^ availability, engages sirtuin- and PARP-mediated signalling, mitochondrial biogenesis, and anabolic flux during organogenesis (Bertoldo et al., 2020; Katsyuba and Auwerx, 2017), which may shift resource allocation toward reproductive investment at the cost of developmental speed – a classic life-history trade-off. That such metabolic reprogramming can redirect tissue-specific developmental programmes without elevating whole-animal RMR highlights the importance of distinguishing between cellular and whole-organism levels of metabolic organisation and is consistent with the broader view that mitochondrial function and RMR are not tightly coupled (Thoral et al., 2024). Future work integrating measures of mitochondrial function with whole-organism metabolism and fitness traits across multiple environments, including nuclear genomes (for an example of how two different nuclear backgrounds impact traits, see Camus et al., 2020), will be necessary to disentangle the mechanistic basis of this apparent decoupling.

## Supplementary Material

10.1242/jexbio.253155_sup1 Supplementary information

## References

[JEB253155C1] Aw, W. C., Towarnicki, S. G., Melvin, R. G., Youngson, N. A., Garvin, M. R., Hu, Y., Nielsen, S., Thomas, T., Pickford, R., Bustamante, S. et al. (2018). Genotype to phenotype: diet-by-mitochondrial DNA haplotype interactions drive metabolic flexibility and organismal fitness. *PLoS Genet.* 14, e1007735. 10.1371/journal.pgen.1007735 PMC6219761 30399141

[JEB253155C2] Bates, D., Mächler, M., Bolker, B. and Walker, S. (2015). Fitting linear mixed-effects models using lme4. *J. Stat. Softw.* 67, 1-48. 10.18637/jss.v067.i01

[JEB253155C3] Bertoldo, M. J., Listijono, D. R., Ho, W.-H. J., Riepsamen, A. H., Goss, D. M., Richani, D., Jin, X. L., Mahbub, S., Campbell, J. M., Habibalahi, A. et al. (2020). NAD+ repletion rescues female fertility during reproductive aging. *Cell Rep.* 30, 1670-1681.e7. 10.1016/j.celrep.2020.01.058 PMC7063679 32049001

[JEB253155C4] Bettinazzi, S., Liang, J., Rodriguez, E., Bonneau, M., Holt, R., Whitehead, B., Dowling, D., Lane, N. and Camus, M. F. (2024). Assessing the role of mitonuclear interactions on mitochondrial function and organismal fitness in natural *Drosophila* populations. *Evol. Lett.* 8, 916-926. 10.1093/evlett/qrae043 PMC11637609 39677574

[JEB253155C5] Bettinazzi, S., Ghirardello, M., Combeaud, A., Fan, J., Bonneau, M., Fowler, K., Dowling, D. K. and Camus, M. F. (2025). Mitonuclear interactions and early-life diet shape adult nutritional behaviour. *J. Evol. Biol.* 38, 1601-1611. 10.1093/jeb/voaf123 41091849

[JEB253155C7] Camus, M. F. and Inwongwan, S. (2023). Mitonuclear interactions modulate nutritional preference. *Biol. Lett.* 19, 20230375. 10.1098/rsbl.2023.0375 PMC10698477 38053364

[JEB253155C8] Camus, M. F., O'Leary, M., Reuter, M. and Lane, N. (2020). Impact of mitonuclear interactions on life-history responses to diet. *Philos. Trans. R. Soc. B* 375, 20190416. 10.1098/rstb.2019.0416 PMC6939373 31787037

[JEB253155C9] Camus, M. F., Rodriguez, E., Kotiadis, V., Carter, H. and Lane, N. (2023). Redox stress shortens lifespan through suppression of respiratory complex I in flies with mitonuclear incompatibilities. *Exp. Gerontol.* 175, 112158. 10.1016/j.exger.2023.112158 36965604

[JEB253155C10] Clancy, D. J. (2008). Variation in mitochondrial genotype has substantial lifespan effects which may be modulated by nuclear background. *Aging Cell* 7, 795-804. 10.1111/j.1474-9726.2008.00428.x 18727704

[JEB253155C11] Dobson, A. J., Voigt, S., Kumpitsch, L., Langer, L., Voigt, E., Ibrahim, R., Dowling, D. K. and Reinhardt, K. (2023). Mitonuclear interactions shape both direct and parental effects of diet on fitness and involve a SNP in mitoribosomal 16s rRNA. *PLoS Biol.* 21, e3002218. 10.1371/journal.pbio.3002218 PMC10441796 37603597

[JEB253155C12] Gyllenhammer, L. E., Entringer, S., Buss, C. and Wadhwa, P. D. (2020). Developmental programming of mitochondrial biology: a conceptual framework and review. *Proc. R. Soc. B* 287, 20192713. 10.1098/rspb.2019.2713 PMC7282904 32345161

[JEB253155C13] Katsyuba, E. and Auwerx, J. (2017). Modulating NAD^+^ metabolism, from bench to bedside. *EMBO J.* 36, 2670-2683. 10.15252/embj.201797135 PMC5599801 28784597

[JEB253155C14] Makwisa, E. M., Procházka, E., Gáliková, M. and Klepsatel, P. (2026). Influence of developmental diet on adult energy reserves in Drosophila. *BMC Biol.* 24, 9. 10.1186/s12915-025-02486-3 PMC12797408 41353133

[JEB253155C15] Meiklejohn, C. D., Holmbeck, M. A., Siddiq, M. A., Abt, D. N., Rand, D. M. and Montooth, K. L. (2013). An Incompatibility between a mitochondrial tRNA and its nuclear-encoded tRNA synthetase compromises development and fitness in *Drosophila*. *PLoS Genet.* 9, e1003238. 10.1371/journal.pgen.1003238 PMC3561102 23382693

[JEB253155C16] Metcalfe, N. B., Bellman, J., Bize, P., Blier, P. U., Crespel, A., Dawson, N. J., Dunn, R. E., Halsey, L. G., Hood, W. R., Hopkins, M. et al. (2023). Solving the conundrum of intra-specific variation in metabolic rate: a multidisciplinary conceptual and methodological toolkit. *BioEssays* 45, e2300026. 10.1002/bies.202300026 37042115

[JEB253155C17] Moran, B. M., Payne, C. Y., Powell, D. L., Iverson, E. N. K., Donny, A. E., Banerjee, S. M., Langdon, Q. K., Gunn, T. R., Rodriguez-Soto, R. A., Madero, A. et al. (2024). A lethal mitonuclear incompatibility in complex I of natural hybrids. *Nature* 626, 119-127. 10.1038/s41586-023-06895-8 PMC10830419 38200310

[JEB253155C18] Mossman, J. A., Tross, J. G., Jourjine, N. A., Li, N., Wu, Z. and Rand, D. M. (2017). Mitonuclear interactions mediate transcriptional responses to hypoxia in Drosophila. *Mol. Biol. Evol.* 34, 447-466. 10.1093/molbev/msw246 PMC6095086 28110272

[JEB253155C31] Patel, M. R., Miriyala, G. K., Littleton, A. J., Yang, H., Trinh, K., Young, J. M., Kennedy, S. R., Yamashita, Y. M., Pallanck, L. J. and Malik, H. S. (2016). A mitochondrial DNA hypomorph of cytochrome oxidase specifically impairs male fertility in Drosophila melanogaster. *eLife* 5, e16923. 10.7554/eLife.16923 PMC4970871 27481326

[JEB253155C19] Piccinini, G., Iannello, M., Puccio, G., Plazzi, F., Havird, J. C. and Ghiselli, F. (2021). Mitonuclear coevolution, but not nuclear compensation, drives evolution of OXPHOS complexes in bivalves. *Mol. Biol. Evol.* 38, 2597-2614. 10.1093/molbev/msab054 PMC8136519 33616640

[JEB253155C20] Quirós, P. M., Mottis, A. and Auwerx, J. (2016). Mitonuclear communication in homeostasis and stress. *Nat. Rev. Mol. Cell Biol.* 17, 213-226. 10.1038/nrm.2016.23 26956194

[JEB253155C21] Rand, D. M. and Mossman, J. A. (2020). Mitonuclear conflict and cooperation govern the integration of genotypes, phenotypes and environments. *Philos. Trans. R. Soc. B* 375, 20190188. 10.1098/rstb.2019.0188 PMC6939372 31787039

[JEB253155C22] Rank, N. E., Mardulyn, P., Heidl, S. J., Roberts, K. T., Zavala, N. A., Smiley, J. T. and Dahlhoff, E. P. (2020). Mitonuclear mismatch alters performance and reproductive success in naturally introgressed populations of a montane leaf beetle. *Evolution* 74, 1724-1740. 10.1111/evo.13962 32246837

[JEB253155C23] Rodríguez, E., Grover Thomas, F., Camus, M. F. and Lane, N. (2021). Mitonuclear interactions produce diverging responses to mild stress in Drosophila Larvae. *Front. Genet.* 12, 734255. 10.3389/fgene.2021.734255 PMC8482813 34603395

[JEB253155C24] Salin, K., Auer, S. K., Rudolf, A. M., Anderson, G. J., Selman, C. and Metcalfe, N. B. (2016). Variation in metabolic rate among individuals is related to tissue-specific differences in mitochondrial leak respiration. *Physiol. Biochem. Zool.* 89, 511-523. 10.1086/688769 27792536

[JEB253155C25] Samuni, Y., Goldstein, S., Dean, O. M. and Berk, M. (2013). The chemistry and biological activities of N-acetylcysteine. *Biochim. Biophys. Acta Gen. Subj.* 1830, 4117-4129. 10.1016/j.bbagen.2013.04.016 23618697

[JEB253155C26] Stefanatos, R., Robertson, F., Castejon-Vega, B., Yu, Y., Uribe, A. H., Myers, K., Kataura, T., Korolchuk, V. I., Maddocks, O. D. K., Martins, L. M. et al. (2025). Developmental mitochondrial complex I activity determines lifespan. *EMBO Rep.* 26, 1957-1983. 10.1038/s44319-025-00416-6 PMC12019323 40097814

[JEB253155C27] Thoral, E., García-Díaz, C. C., Persson, E., Chamkha, I., Elmér, E., Ruuskanen, S. and Nord, A. (2024). The relationship between mitochondrial respiration, resting metabolic rate and blood cell count in great tits. *Biol. Open* 13, bio060302. 10.1242/bio.060302 PMC10958200 38385271

[JEB253155C28] Thoral, E., Nati, J., Quémeneur, J.-B., Lozac'h, L., Salou, G., Vergnet, A., Besson, M., Vandeputte, M., Allal, F., Salin, K. et al. (2026). Mitochondrial respiratory capacities are not linked to individual variation in hypoxia tolerance and standard metabolic rate in European seabass *Dicentrarchus labrax*. *J. Fish Biol.* 109, 1086-1094. 10.1111/jfb.70491 PMC13521724 42126105

[JEB253155C29] Wolff, J. N., Ladoukakis, E. D., Enríquez, J. A. and Dowling, D. K. (2014). Mitonuclear interactions: evolutionary consequences over multiple biological scales. *Philos. Trans. R. Soc. B Biol. Sci.* 369, 20130443. 10.1098/rstb.2013.0443 PMC4032519 24864313

[JEB253155C30] Wong, C.-O., Li, R., Montell, C. and Venkatachalam, K. (2012). *Drosophila* TRPML is required for TORC1 activation. *Curr. Biol.* 22, 1616-1621. 10.1016/j.cub.2012.06.055 PMC3443270 22863314

