# Supplementary material for: Mitochondrial genetic effects mediate the response to stress through development, but not adult metabolic rate in Drosophila melanogaster
Source: J Exp Biol. 2026 Oct 20;229(20):jeb253155. doi: 10.1242/jeb.253155 (PMC13723412; doi:10.1242/jeb.253155)
Supplement: Supplementary file 1 [file jexbio-229-253155-s1.pdf]

Table S1. Development and survival analysis across mitonuclear lines and treatment. Data refer to Fig. 1.

1. DEVELOPMENT TIME x TREATMENT ANALYSIS			
**Line**	**Treatment**	**Mean ± SEM**	**Median**
WT	Control	256.77 ± 0.527	251
BAR	Control	253.85 ± 0.376	251
COX	Control	257.79 ± 0.626	251
WT	High Protein	245.08 ± 0.412	247
BAR	High Protein	247.88 ± 0.436	247
COX	High Protein	248.46 ± 0.528	247
WT	NAC	270.40 ± 0.619	271
BAR	NAC	272.02 ± 0.500	271
COX	NAC	298.43 ± 0.618	299
WT	NR	260.63 ± 0.889	251
BAR	NR	259.30 ± 0.612	251
COX	NR	259.00 ± 0.600	251

**ANOVA**	final model	Time ~ Line* Sex*Treatment *(1|Batch)
	:Line	**X^2=335.95, *P* = 2.2e-16 *****
	:Sex	**X^2= 46.97, *P* = 7.2e-12 *****
	:Treatment	**X^2= 5256.36, *P* = 2.2e-16 *****
	Line:Sex	**X^2= 9.68, *P* = 0.008 ****
	Line:Treatment	**X^2= 1040.45, *P* = 2.2e-16 *****
	Sex:Treatment	**X^2= 42.61, *P* = 3.0e-09 *****
	Line:Sex:Treatment	X^2= 1.9504, *P* = 0.92

Females' DEVELOPMENT TIME x TREATMENT ANALYSIS							
**Treatment**	**Contrast**	**Estimate**	**SE**	**df**	**t-value**	**p-value**	**Sig**
C	WT - COX	-1.426	1.1	2927	-1.293	0.3994	ns
C	WT - BAR	2.796	1.07	2927	2.616	0.0243	*
C	COX - BAR	4.222	1.12	2927	3.773	0.0005	***
NAC	WT - COX	-29.445	1.25	2927	-23.477	<.0001	***
NAC	WT - BAR	-3.313	1.2	2927	-2.754	0.0163	*
NAC	COX - BAR	26.132	1.17	2927	22.268	<.0001	***
HP	WT - COX	-3.158	1.09	2927	-2.901	0.0105	*
HP	WT - BAR	-2.108	1.04	2927	-2.021	0.1072	ns
HP	COX - BAR	1.05	1.07	2927	0.983	0.5876	ns
NR	WT - COX	-1.108	1.09	2927	-1.014	0.5679	ns
NR	WT - BAR	-0.611	1.18	2927	-0.52	0.8617	ns
NR	COX - BAR	0.498	1.16	2927	0.429	0.9037	ns

Females' dev time post-hoc

**Contrasts / Line**	**BAR**	**COX**	**WT**
C-HP	**7.85, p=<.0001**	**11.02, p=<.0001**	**12.75, p=<.0001**
C-NAC	**-19.15, p=<.0001**	**-41.06, p=<.0001**	**-13.04, p=<.0001**
C-NR	**-7.59, p=<.0001**	**-3.86, p=0.0030**	**-4.18, p=0.0006**
HP-NAC	**26.99, p=<.0001**	**52.08, p=<.0001**	**25.79, p=<.0001**
HP-NR	**-15.44, p=<.0001**	**-13.58, p=<.0001**	**-16.93, p=<.0001**
NAC-NR	**11.56, p=<.0001**	**37.19, p=<.0001**	**8.85, p=<.0001**

**Contrast / Treat**	**Control**	**High Protein**	**NAC**	**NR**
BAR-COX	**4.22, p=0.0005**	1.05, p=0.58	**26.44, p=<.0001**	0.49, p=0.90
BAR-WT	**2.79, p=0.024**	-2.108, p=0.107	**-3.33, p=0.02**	-0.61, p=0.86
COX- WT	-1.43, p= 0.39	**-3.15, p=0.010**	**-29.44, p=<.0001**	-1.108, p=0.56

Males' dev time post-hoc

**Contrasts**	**BAR**	**COX**	**WT**
C-HP	**4.30, p=0.0003**	**8.25, p=<.0001**	**9.69, p=<.0001**
C-NAC	**-16.93, p=<.0001**	**-38.40, p=<.0001**	**-11.99, p=<.0001**
C-NR	**-5.30, p=<.0001**	-0.0833, p=0.99	**-4.06, p=0.0006**
HP-NAC	**21.24, p=<.0001**	**46.66, p=<.0001**	**21.69, p=<.0001**
HP-NR	**-9.61, p=<.0001**	**-8.33, p=<.0001**	**-13.76, p=<.0001**
NAC-NR	**11.62, p=<.0001**	**38.32, p=<.0001**	**7.92, p=<.0001**

**2. SURVIVAL x TREATMENT ANALYSIS**		
**Line**	**Treatment**	**Mean ± SEM**
WT	Control	0.846 ± 0.028
BAR	Control	0.806 ± 0.020
COX	Control	0.780 ± 0.029
WT	High Protein	0.829 ± 0.034
BAR	High Protein	0.828 ± 0.027
COX	High Protein	0.669 ± 0.056
WT	NAC	0.814 ± 0.029
BAR	NAC	0.784 ± 0.035
COX	NAC	0.770 ± 0.043
WT	NR	0.759 ± 0.052
BAR	NR	0.661 ± 0.039
COX	NR	0.766 ± 0.032

**ANOVA**	final model	Prop_survival ~ Line*Treatment +(1|Batch)
	:Line	**X^2= 6.58, *P* = 0.037 ***
	:Treatment	**X^2= 9.88, *P* = 0.020 ***
	Line:Treatment	X^2= 12.1988, *P* = 0.058 .

**Contrasts / Line**	**BAR**	**COX**	**WT**
C-HP	-0.024, p=0.95	**0.13, p=0.04**	0.003, p=0.99
C-NAC	0.017, p=0.97	0.035, p=0.85	0.021, p=0.97
C-NR	**0.14, p=0.006**	-0.026, p=0.95	0.09, p=0.14
HP-NAC	0.04, p=0.82	-0.92, p=0.25	0.018, 0.99
HP-NR	**0.16, p=0.002**	**-0.15, p=0.024**	0.092, p=0.18
NAC-NR	**0.122, p=0.03**	-0.062, p=0.61	0.074, p=0.38

**Contrasts / Treatment**	**Control**	**High Protein**	**NAC**	**NR**
BAR-COX	0.026, p=0.84	**0.178, p=0.001**	0.044, p=0.59	**-0.14, p=0.01**
BAR-WT	-0.035, p=0.69	-0.008, p=1	-0.03, p=0.78	-0.08, p=0.17
COX- WT	-0.061, p=0.33	**-0.186, p=0.0007**	-0.07, p=0.24	0.06, p=0.45

Journal of Experimental Biology • Supplementary information

Table S2. Female fitness and adult body mass analysis across mitonuclear lines and treatments. Data refer to Fig. 2.

**TOTAL OFFSPRING**		
1. TOTAL OFFSPRING x TREATMENT ANALYSIS		
**Line**	**Treatment**	**Mean ± SEM**
WT	Control	112.18 ± 9.17
BAR	Control	72.03 ± 9.12
COX	Control	68.89 ± 8.13
WT	High Protein	81.63 ± 8.65
BAR	High Protein	93.84 ± 7.32
COX	High Protein	108.51 ± 8.66
WT	NAC	101.40 ± 6.73
BAR	NAC	110.77 ± 7.67
COX	NAC	112.94 ± 8.22
WT	NR	121.76 ± 6.85
BAR	NR	116.05 ± 7.63
COX	NR	121.60 ± 8.25

**ANOVA**	final model	glm.nb(Total_offspring ~ Treatment * Line)
	:Treatment	**X^2= 21.62, *P* = *7.828*e-05 *****
	:Line	X^2= 0.96, *P* = 0.619
	Line:Treatment	**X^2= 18.69, *P* = 0.004 ****

Female total offspring post-hoc

**Contrasts / Line**	**BAR (Ratio, SE, z.ratio, p)**	**COX (Ratio, SE, z.ratio, p)**	**WT (Ratio, SE, z.ratio, p)**
C-HP	0.768, 0.110, -1.850, p = 0.250	**0.635, 0.089, -3.251, p = 0.0063**	1.374, 0.195, 2.245, p = 0.111
C-NAC	**0.650, 0.095, -2.959, p = 0.0163**	**0.610, 0.086, -3.513, p = 0.0025**	1.106, 0.156, 0.715, p = 0.891
C-NR	**0.621, 0.090, -3.301, p = 0.0053**	**0.566, 0.079, -4.069, p = 0.0003**	0.921, 0.128, -0.588, p = 0.936
HP-NAC	0.847, 0.116, -1.214, p = 0.618	0.961, 0.135, -0.285, p = 0.992	0.805, 0.112, -1.554, p = 0.406
HP-NR	0.809, 0.110, -1.567, p = 0.397	0.892, 0.124, -0.818, p = 0.846	**0.670, 0.092, -2.907, p=0.019**
NAC-NR	0.954, 0.132, -0.337, p = 0.987	0.929, 0.130, -0.527, p = 0.953	0.833, 0.114, -1.332, p = 0.542

**Contrast / Treat**	**Control (Ratio, SE, z.ratio, p)**	**High Protein(Ratio, SE, z.ratio, p)**	**NAC (Ratio, SE, z.ratio, p)**	**NR (Ratio, SE, z.ratio, p)**

BAR-COX	1.046, 0.153, 0.306, p = 0.950	0.865, 0.118, -1.063, p = 0.537	0.981, 0.138, -0.138, p = 0.990	0.954, 0.132, -0.338, p = 0.939
BAR-WT	**0.642, 0.095, -3.005, p = 0.0075**	1.150, 0.157, 1.018, p = 0.565	1.092, 0.152, 0.635, p = 0.801	0.953, 0.130, -0.352, p = 0.934
COX- WT	**0.614, 0.087, -3.438, p = 0.0017**	**1.329, 0.186, 2.040, p = 0.103**	1.114, 0.156, 0.768, p = 0.722	0.999, 0.137, -0.009, p = 1.000

**2. BODY MASS x TREATMENT ANALYSIS**			
**Line**	**Treatment**	**Female Mean ± SEM**	**Male Mean ± SEM**
WT	Control	6.34 ± 0.13	3.86 ± 0.079
BAR	Control	5.86 ± 0.24	3.62 ± 0.085
COX	Control	5.86 ± 0.19	3.78 ± 0.054
WT	High Protein	6.89 ± 0.23	4.12 ± 0.043
BAR	High Protein	6.70 ± 0.19	4.11 ± 0.055
COX	High Protein	6.93 ± 0.15	4.20 ± 0.073
WT	NAC	6.68 ± 0.18	3.84 ± 0.048
BAR	NAC	6.29 ± 0.18	3.81 ± 0.037
COX	NAC	6.38 ± 0.19	3.87 ± 0.063
WT	NR	6.61 ± 0.23	3.88 ± 0.049
BAR	NR	6.54 ± 0.21	3.89 ± 0.038
COX	NR	6.08 ± 0.23	3.77 ± 0.096

**ANOVA**	final model	mass ~ line*(1|age)*sex*treatment+(1|block)
	:Line	**X^2= 8.65, *P* = 0.013 ***
	:Sex	**X^2=2137.96, *P* = 2.2e-16 *****
	:Treatment	**X^2= 63.76, *P* = 9.245e-14 *****
	Line:Sex	X^2= 5.24, *P* = 0.073 .
	Line:Treatment	X^2= 9.97, *P* = 0.126
	Sex:Treatment	**X^2= 8.65, *P* = 0.034 ***
	Line:Sex:Treatment	X^2= 2.45, *P* = 0.874

Females' mass post-hoc

**Contrasts / Lines**	**BAR (Estimate, SE, t.ratio, p)**	**COX (Estimate, SE, t.ratio, p)**	**WT (Estimate, SE, t.ratio, p)**
C-HP	**-0.903, 0.256, -3.522, p = 0.0035**	**-1.111, 0.255, -4.358, p = 0.0002**	-0.572, 0.255, -2.244, p = 0.1180
C-NAC	-0.443, 0.259, -1.710, p = 0.3234	-0.537, 0.262, -2.050, p = 0.1764	-0.340, 0.262, -1.299, p = 0.5654

C-NR	**-0.704, 0.262, -2.689, p = 0.0407**	-0.220, 0.259, -0.849, p = 0.8310	-0.289, 0.255, -1.132, p = 0.6705
HP-NAC	0.460, 0.256, 1.794, p = 0.2819	0.574, 0.256, 2.240, p = 0.1191	0.232, 0.256, 0.904, p = 0.8026
HP-NR	0.199, 0.255, 0.780, p = 0.8635	**0.891, 0.255, 3.496, p = 0.0038**	0.283, 0.247, 1.147, p = 0.6612
NAC-NR	-0.261, 0.262, -0.997, p = 0.7514	0.317, 0.262, 1.210, p = 0.6218	0.052, 0.256, 0.201, p = 0.9971

**Contrasts / Treatment**	**Control (Estimate, SE, t.ratio, p)**	**High Protein (Estimate, SE, t.ratio, p)**	**NAC (Estimate, SE, t.ratio, p)**	**NR (Estimate, SE, t.ratio, p)**
BAR-COX	-0.014, 0.262, -0.052, p = 0.999	-0.222, 0.247, -0.898, p = 0.643	-0.107, 0.259, -0.414, p = 0.910	0.471, 0.259, 1.817, p = 0.169
BAR-WT	-0.518, 0.262, -1.977, p = 0.123	-0.187, 0.247, -0.756, p = 0.731	-0.415, 0.259, -1.601, p = 0.250	-0.102, 0.255, -0.400, p = 0.916
COX- WT	-0.504, 0.259, -1.945, p = 0.131	0.035, 0.247, 0.142, p = 0.989	-0.307, 0.259, -1.187, p = 0.464	-0.573, 0.255, -2.247, p = 0.068

Males' mass post-hoc

**Contrasts / Lines**	**BAR (Estimate, SE, t.ratio, p)**	**COX (Estimate, SE, t.ratio, p)**	**WT (Estimate, SE, t.ratio, p)**
C-HP	**-0.473, 0.075, -6.329, p < 0.0001**	**-0.428, 0.074, -5.753, p < 0.0001**	**-0.265, 0.074, -3.568, p = 0.0029**
C-NAC	**-0.190, 0.075, -2.521, p = 0.0619**	-0.115, 0.076, -1.512, p = 0.4337	-0.002, 0.076, -0.033, p = 1.0000
C-NR	**-0.247, 0.076, -3.236, p = 0.0084**	0.014, 0.075, 0.181, p = 0.9979	-0.028, 0.074, -0.372, p = 0.9823
HP-NAC	**0.283, 0.075, 3.787, p = 0.0014**	**0.312, 0.075, 4.180, p = 0.0003**	**0.263, 0.075, 3.515, p = 0.0034**
HP-NR	**0.226, 0.074, 3.047, p = 0.0149**	**0.441, 0.074, 5.936, p < 0.0001**	**0.238, 0.072, 3.291, p = 0.0071**
NAC-NR	-0.057, 0.076, -0.743, p = 0.8795	0.129, 0.076, 1.691, p = 0.3331	-0.025, 0.075, -0.337, p = 0.9867

Journal of Experimental Biology • Supplementary information

**Contrasts / Treatment**	**Control (Estimate, SE, t.ratio, p)**	**High Protein (Estimate, SE, t.ratio, p)**	**NAC (Estimate, SE, t.ratio, p)**	**NR (Estimate, SE, t.ratio, p)**
BAR-COX	-0.133, 0.076, -1.745, p = 0.193	-0.088, 0.072, -1.212, p = 0.448	-0.058, 0.075, -0.772, p = 0.721	**0.127, 0.075, 1.688, p = 0.214**
BAR-WT	**-0.212, 0.076, -2.783, p = 0.017**	-0.004, 0.072, -0.058, p = 0.998	-0.025, 0.075, -0.326, p = 0.943	0.007, 0.074, 0.092, p = 0.995
COX- WT	-0.079, 0.075, -1.049, p = 0.548	0.083, 0.072, 1.155, p = 0.483	0.034, 0.075, 0.446, p = 0.896	-0.120, 0.074, -1.620, p = 0.241

Table S3. Resting metabolic rate and activity analysis across mitonuclear lines and treatments. Data refer to Fig. 3.

**METABOLIC RATE**				
			**Males (M)**	**Females (F)**
**ANOVA**	final model		lmer (logCO2 ~ line*treatment +(1|block)+(1|run)+meanM/F.logmass+meanM/F. activity)	
	:Treatment		**X^2= 7.86, p = 0.048***	X^2= 0.889, *P* = *0.82*
	:Line		X^2= 1.37, p = 0.50	X^2= 3.31, *P* = 0.191
	Line:Treatment		X^2= 0.89, *P* = 0.98	X^2= 1.705, *P* = 0.636
			Tukey-adjusted pairwise comparisons did not identify significant differences between individual treatment groups.	
**ACTIVITY**				
**ANOVA**	final model		lmer(activity ~line*treatment +(1|block) +(1|run) +chamber+weight)	
	:Treatment		X^2= 0.889, p = 0.828	X^2= 4.66, p = 0.198
	:Line		X^2= 3.31, p = 0.191	X^2= 2.868, *P* = 0.238
	Line:Treatment		X^2= 1.705, *P* = 0.63	X^2= 0.90, *P* = 0.82

Journal of Experimental Biology • Supplementary information

Table S4. *Drosophila* food recipes showing the composition of the four treatments (control, high protein - HP, NAC, NR). Agar serves as the gelling base, water as the solvent, and sugar and yeast amounts are modified to regulate carbohydrate and protein content. Nipagin and propionic acid act as preservatives. Y:C denotes the yeast-to-carbohydrate ratio.

**Compound / Diet**	**Control diet**	**High Protein diet**	**NAC 5 g/L**	**NR 10 mM**
Y:C	1:4	1:2	1:4	1:4
Agar	15 g	15 g	15 g	15 g
H_2O (total)	1 L	1 L	1 L	1 L
Molasses	200 mL	80 mL	200 mL	200 mL
Yeast	27.33 g	107.33 g	27.33 g	27.33 g
Nipagin 10%	30 mL	30 mL	30 mL	30 mL
Propionic Acid	3 mL	3 mL	3 mL	3 mL
Other	-	-	5 g N-acetyl-L-cysteine (Sigma A7250)	2.552 g of Nicotinamide Riboside (CAS 1341-23-7)
